# A web-based tool to predict acute kidney injury in patients with ST-elevation myocardial infarction: Development, internal validation and comparison

**DOI:** 10.1371/journal.pone.0181658

**Published:** 2017-07-31

**Authors:** Benjamin R. Zambetti, Fridtjof Thomas, Inyong Hwang, Allen C. Brown, Mason Chumpia, Robert T. Ellis, Darshan Naik, Rami N. Khouzam, Uzoma N. Ibebuogu, Guy L. Reed

**Affiliations:** 1 Department of Medicine, University of Tennessee Health Science Center, Memphis, Tennessee, United States of America; 2 Department of Preventive Medicine, University of Tennessee Health Science Center, Memphis, Tennessee, United States of America; University of Messina, ITALY

## Abstract

**Background:**

In ST-elevation myocardial infarction (STEMI), acute kidney injury (AKI) may increase subsequent morbidity and mortality. Still, it remains difficult to predict AKI risk in these patients. We sought to 1) determine the frequency and clinical outcomes of AKI and, 2) develop, validate and compare a web-based tool for predicting AKI.

**Methods & findings:**

In a racially diverse series of 1144 consecutive STEMI patients, Stage 1 or greater AKI occurred in 12.9% and was severe (Stage 2–3) in 2.9%. AKI was associated with increased mortality (5.7-fold, unadjusted) and hospital stay (2.5-fold). AKI was associated with systolic dysfunction, increased left ventricular end-diastolic pressures, hypotension and intra-aortic balloon counterpulsation. A computational algorithm (UT-AKI) was derived and internally validated. It showed higher sensitivity and improved overall prediction for AKI (area under the curve 0.76) vs. other published indices. Higher UT-AKI scores were associated with more severe AKI, longer hospital stay and greater hospital mortality.

**Conclusions:**

In a large, racially diverse cohort of STEMI patients, Stage 1 or greater AKI was relatively common and was associated with significant morbidity and mortality. A web-accessible, internally validated tool was developed with improved overall value for predicting AKI. By identifying patients at increased risk, this tool may help physicians tailor post-procedural diagnostic and therapeutic strategies after STEMI to reduce AKI and its associated morbidity and mortality.

## Introduction

Worldwide, ischemic heart disease is responsible for the greatest number of years of life lost [1]. ST-elevation myocardial infarction (STEMI) is a major cause of acute morbidity and mortality in patients with ischemic heart diseases. Where available, the standard of care treatment for STEMI is diagnostic angiography followed by percutaneous coronary intervention (PCI), which is designed to restore coronary blood flow rapidly. However, STEMI patients may develop acute kidney injury (AKI) whether or not they receive PCI. Identifying patients at risk could help physicians mitigate the morbidity and mortality of AKI.

The risk of AKI has been examined in patients undergoing elective or non-urgent diagnostic angiography or PCI [2] and several risk factors have been identified [3–9]. By comparison, to elective angiography, few studies have attempted to identify variables specifically associated with the risk of AKI in patients undergoing PCI for STEMI. Limited data suggests that STEMI patient may have as much as a four-fold higher risk of developing renal injury than patients with stable coronary artery disease, possibly due to concomitant hypotension, altered cardiac output, systemic thromboembolism, and other factors [9–11]. In addition, there is little information about the relationship between risk factors and the severity of renal injury. Recent studies suggest that beyond the traditional definitions of contrast nephropathy, the severity of AKI should be categorized according to criteria that reflect the impact of renal injury on meaningful clinical outcomes. For example, the Risk, Injury, Failure, Loss, End Stage kidney disease criteria (RIFLE) categorize renal injury into six groups (risk, injury, failure, loss, end stage kidney disease) [12]. The AKI Network classification system identifies three increasing stages of renal injury (Stages 1–3) [13].

The goal of this study was to predict the development and severity of AKI, defined by modified RIFLE and AKI Network criteria, and to determine its effects on short-term morbidity and mortality. A web-based tool (UT-AKI index, https://www.uthsc.edu/cardiology/research/utaki.php) was developed and internally validated to predict risk of AKI in a large, consecutive, racially diverse cohort of patients undergoing diagnostic angiography and PCI for acute STEMI. The UT-AKI index showed significant associations with the severity of AKI, hospital stay and mortality. The UT-AKI index also significantly enhanced the prediction of STEMI patients at risk for AKI, which may allow physicians to mitigate the morbidity and mortality associated with renal injury, by reducing exposure to other agents with known renal toxicity and, by tailoring post-procedural monitoring of kidney function to potential risk.

## Methods

The development of AKI was examined in consecutive patients who were referred by emergency physicians for primary PCI treatment of STEMI to the UT Methodist Hospital between January 2008 and September 2013. Patients undergoing dialysis were excluded from the analyses. This study was approved by the University of Tennessee Health Sciences Institutional Review Board as exempt from the informed consent requirement under U.S. 45CFR46.101(b)(4), as it used data in a manner in which subjects could not be individually identified.

### Data collection

Data were obtained from medical records corresponding to the hospitalization for STEMI using a prospectively designed collection tool with pre-defined criteria. Demographic variables such as sex, race and marital status were self-reported and were retrieved from hospital registration records. Patient’s past medical history (family history of coronary artery disease, diabetes, previous myocardial infarction, heart failure, hypertension, hyperlipidemia, chronic kidney disease, smoking, ethanol use, body mass index) were obtained from the initial evaluation in the emergency room. Procedural characteristics (PCI, coronary bypass surgery, coronary stenoses, contrast volume, hypotension, intra-aortic balloon pump) and hemodynamic data (left ventricular ejection fraction and end diastolic pressure) were obtained from cardiology documents. Laboratory values (creatinine, hemoglobin, troponin) were obtained from laboratory records. Left ventricular ejection fraction was obtained by left ventriculography, echocardiography or the average of the two measures if both were available. Hypotension was defined as systolic blood pressure < 80 mmHg as specified by Mehran, et al [7]. Anemia was defined as a hemoglobin <13 g/dL for men and <12 g/dL for women [14]. All of these variables were included in initial univariate analysis.

### AKI classification

The severity of AKI after PCI was graded by changes in serum creatinine and estimated GFR (eGFR) by the MDRD method[15] within 3–5 days post-STEMI using criteria similar to the RIFLE and AKIN indices for acute kidney injury. Stage 1 AKI corresponded to Risk in the RIFLE index and was defined by an increase in serum creatinine of 1.5-fold or a decrease in eGFR of >25%. Stage 2 corresponded to the Injury classification of the RIFLE system: a 2-fold increase of serum creatinine or an eGFR decrease of >50%. Stage 3 corresponded to Failure in the RIFLE system and was characterized by a rise in serum creatinine of 3-fold, or a serum creatinine ≥ 4 (with a creatinine increase of 0.5 mg/dL) or a decrease in eGFR of >75% [12, 13]. For comparison we also examined AKI defined by traditional criteria of contrast-induced nephropathy, e.g., a 25% relative increase or 0.5 mg/dL increase in serum creatinine from baseline.

### UT-AKI predictive index development

The data set was randomly split once into two equal size groups of 525 patients each using Statistical Package for the Social Sciences (IBM’s SPSS 22 for Mac 2014). These constituted the development and validation data sets, which used for model selection/fitting and model testing, respectively. In the development set, a univariate analysis was performed to identify risk factors showing a significant relationship with the development of AKI (see Data Collection above). These variables and additional established risk factors directly related to renal function were evaluated in multivariable logistic regression. The full multivariate model with all identified variables was subsequently reduced by a backward selection procedure utilizing a threshold of (marginal) p-value of 0.10 to exclude variables in iterative steps (only main effects were considered). The model yieled a UT-AKI score, which is the (predicted) probability that an individual patient will develop AKI based on the identified multivariable logistic regression model. These probabilities can be computed from the parameter estimates as specified in S1 Table. ROC-analysis was used to evaluate the area under the curve (AUC or C-statistic), as well as the sensitivity and specificity obtained when certain thresholds were applied to classify patients predicted to develop AKI (see S1 Text).[16] Goodness of fit for the model was assessed with the Hosmer-Lemeshow statistic. Since ROC analyses required complete data for each of the four risk scores, 117 patients missing one or more variables were excluded from the development data set, leaving 408 patients included for predictive model building.

### Risk score validation

The formula for the UT-AKI risk score obtained from the derivation data set was applied to the patients in the validation data set and a score (predictive probability based on the logistic regression model) was determined for each patient. ROC curves and associated AUCs were produced based on the performance of all risk-scores in the validation data set. Similar to the derivation set, the validation set was missing data required for at least one of the compared indices for 93 patients, resulting in 432 patients utilized in the ROC analyses.

### Risk score calculations

ACEF, AGEF, Mehran, McCullough, NCDR and UT-AKI risk scores were calculated for each patient as described (S1 Table). Every attempt was made to use variables that closely matched those described in the respective original studies/publications.

### Data analysis

Differences between two groups were assessed by Student’s t-test, Mann-Whitney U, or Chi-squared as appropriate. Differences between more than two groups were analyzed by analysis of variance with a Kruskal-Wallis test. The overall probability for correct classification of patients was assessed by analyses of ROC curves by the Mann Whitney U statistic. Patients who did not develop AKI were considered the reference or control group. Statistical analyses were done using IBM’s SPSS 22 for Mac (2014) as well as in R (version 3.2.3).

## Results

**Fig 1:**
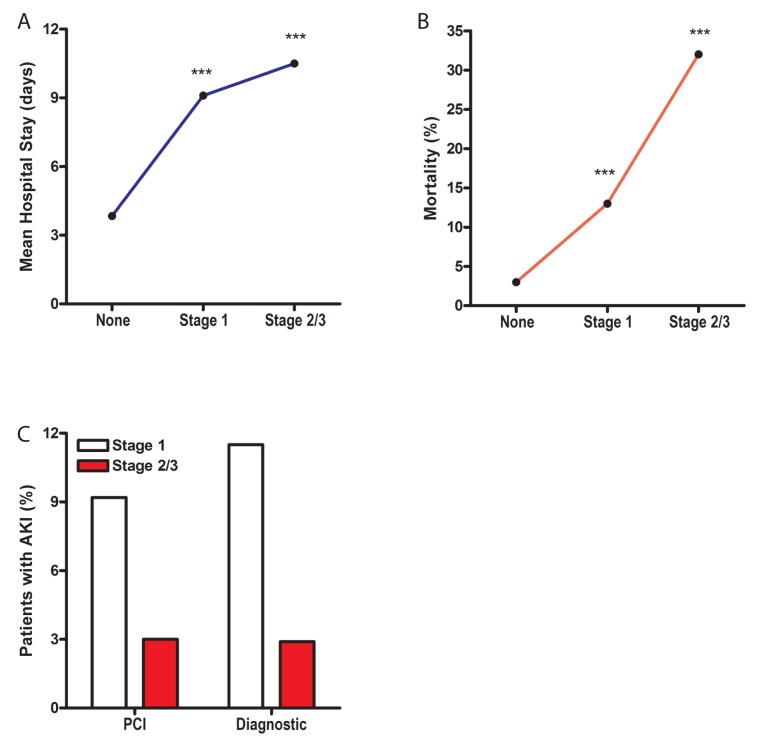
Mortality, length of stay and severity of AKI. A) Mean hospital stay according to the severity of AKI as indicated by stage. B) In-hospital mortality by Stage of AKI. C) Frequency of AKI stage in patients with diagnostic coronary angiography (diagnostic) alone or with PCI. AKI = contrast-induced acute kidney injury, PCI = percutaneous coronary intervention. ***p<0.001 vs. patients with no AKI (none).

During the period of study, 1,144 patients were referred for treatment of STEMI. Of these, 94 patients were excluded from analyses because they were receiving dialysis or did not proceed to diagnostic catheterization or PCI. The mean age of patients was 58.2 years (Table 1). Most patients were male (67%) and African-American (55%). There were no significant differences in the frequency of AKI related to race or sex. Seventy percent underwent PCI. Patients who developed at least Stage 1 AKI (increase of creatinine of 1.5-fold or a decrease in eGFR of >25%), had a 2.5-fold longer hospital stay (Fig 1A, p<0.001). Hospital stay increased with the Stage or severity of AKI: Stage 1 patients stayed an average of 2.4-times longer in the hospital than those who did not develop AKI (3.8 vs. 9.1 days; p≤0.001). Patients with Stage 2/3 stayed 2.8 times longer (3.8 days vs. 10.5 days; p≤0.001). Patients developing Stage 1 or greater AKI had an unadjusted, 5.7-fold increase in mortality vs. patients without renal injury (Fig 1B, p<0.001). Mortality was higher in patients with more severe Stage 2/3 AKI than patients with Stage 1 (32% vs.13%, p<0.001). In contrast, patients who developed ‘contrast-induced nephropathy’ (a 25% relative or a 0.5 mg/dL absolute increase in creatinine), without Stage 1 or greater AKI, did not show a significant increase in length of stay (p = 0.12) or in mortality (p = 0.69).

**Table 1:** Demographics of the study sample.

Variable	Derivation Data Set*	Validation Data Set
**Mean age (years)**	58.2	58.9
**Male, N (%)**	352 (67%)	357, (68%)
**African American, N (%)**	289 (55%)	294 (56%)
**PCI, N (%)**	368 (70%)	373 (71%)
**AKI, N (%)**	68 (13%)	68 (13%)

*The Derivation and Validation Data Sets are 50% random samples of the entire dataset.

Stage 1 or greater AKI occurred in 12.9% of patients overall (Fig 1C). Of these, a small number of patients had Stage 2 (23) or Stage 3 (8) AKI. In patients undergoing PCI the rate of AKI was 12.2%; of these 9.2% had Stage 1, 3.0% had Stage 2/3. In patients undergoing diagnostic coronary angiography alone the rate of AKI was 14.4%. Of these 11.5% had Stage 1 and 2.9% had Stage 2/3. Overall, the frequency of AKI (p = 0.16) and the severity of AKI (p = 0.13) were not significantly different between patients who had diagnostic angiography with or without PCI. There were no significant differences in the type of non-ionic radiographic contrast (Optiray and Visipaque) or the volume of radiocontrast used in those with or without AKI (159 vs. 164 ml).

Cardiac and renal biomarkers were compared for patients at each stage of AKI (Table 2). When compared to patients without AKI, the mean left ventricular ejection fraction (EF) was higher in those without AKI vs. those with Stage 1 or Stage 2/3 AKI. Similarly, the mean left ventricular end diastolic pressure (LVEDP) was significantly lower in those without AKI than in those who developed Stage 1 and Stage 2/3 AKI. The initial mean eGFR was significantly lower in those without AKI compared to Stage 1 patients. However, the mean eGFR of Stage 2/3 was significantly lower than those who did not develop AKI. Patients with Stage 1 or Stage 2/3 AKI had significantly higher troponins and were more likely to receive intra-aortic balloon pump therapy than patients without AKI.

**Table 2:** Cardiac and renal indices in patients with AKI.

Measure	AKI		
	None	Stage 1	Stage 2/3
**LVEF (mean % ± SD)**	48 ± 12%	44 ± 13%***	37 ± 14%***
**LVEDP mm Hg (mean ± SD)**	23 ± 12	27 ± 16***	29 ± 13**
**IABP % (N)**	9% (79)	30% (31)***	36% (11)***
**Hypotension % (N)**	5% (43)	13% (14)***	28% (8)***
**Peak Troponin-I, ng/mg (mean ± SD)**	14 ± 24	24 ± 32***	23 ± 30*
**Initial eGFR ml/min. (mean ± SD)**	77 ± 28	88 ± 43***	58 ± 36***
**Change in eGFR (%, mean ± SD)**	2 ± 14	35 ± 7***	63 ± 14***

*p<0.05

** p<0.01

***p<0.001 compared to no AKI (none) as control.

**Fig 2:**
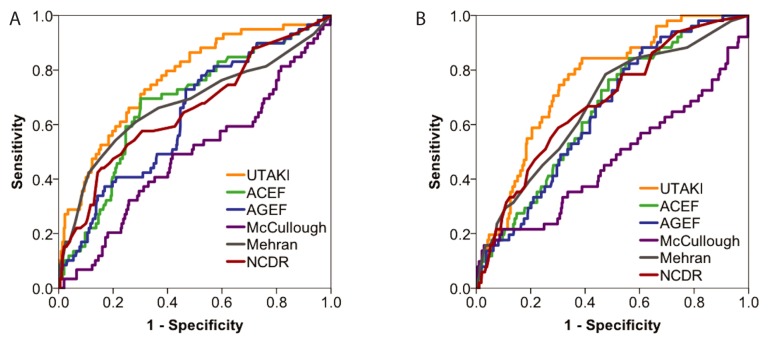
Classification of patients at risk for AKI. ROC curves for A) derivation and B) validation datasets. AUC = area under the curver, ROC = receiver operator characteristic.

Since the development of Stage 1 or greater AKI was associated with clinically significant morbidity and mortality, we sought to derive an index (UT-AKI index) for predicting this risk in STEMI patients. This UT-AKI index was derived using a development data set, randomly selected from half of the patients in our series (see Methods, UT-AKI Predictive Index Development). The variables used in the derived UT-AKI index were: age, history of CKD, eGFR, LVEF, LVEDP and whether the patient was hypotensive and/or received an IABP. The UT-AKI index had an acceptable fit to the data (Hosmer-Lemeshow statistic chi-squared 3.72, p = 0.88). We assessed the performance of the UT-AKI vs. other published indices for predicting Stage 1 or greater AKI by comparing the receiver operating curves (ROC; Fig 2A, Table 3). Overall, the UT-AKI index set showed a larger AUC or C-statistic (0.77) than other indices. To assess the generalizability of these prediction rules, we compared the UT-AKI to other models using the randomly selected validation data set, which had not been used in model development. The ROC curves (Fig 2B, Table 3) were similar to the ROC curves obtained in the development set: The AUC for UT-AKI score was 0.76, which was greater than the AUC for other indices. The UT-AKI showed a similar pattern of prediction of AKI in those undergoing PCI (AUC 0.76) vs. diagnostic angiography alone (AUC 0.76). The performance of these indices in terms of sensitivity and specificity was compared using published score thresholds. Using a UT-AKI threshold score of 0.1 for prediciting AKI in the derivation set, the sensitivity of the UT-AKI score was 80% and the specificity was 60% (Table 4 and S2 Table). By comparison, the sensitivity of all other classifiers was lower. However, the ACEF and NCDR, while having substantially lower sensitivity with 59% and 28%, respectively, had higher specificity (76% and 70%, respectively). The validation data set provided an assessment of the potential generalizability of these prediction rules. In the validation set, the same scoring criteria were applied with the classification scores. For the UT-AKI score, the sensitivity was 84% and the specificity was 61% (Table 4). Again, the sensitivity of the UT-AKI appeared higher than the other indices; the ACEF and NCDR continued to have lower sensitivities (49% and 59%, respectively) but higher specificities (68% and 67%, respectively). The classification performance of these algorithms was also examined by the Kolmogorov-Smirnov threshold or Youden’s index.[17] By these analyses, the UT-AKI index continued to display a pattern of both higher sensitivity and specificity vs. other indices (S3 Table).

**Table 3:** Area under the ROC curve (AUC) for predictive indices in the derivation and valdation data sets.

Predictive Index	AUC (95% CI*) Derivation Data Set		AUC (95% CI) Validation Data Set	
**UT-AKI**	0.77	(0.70, 0.83)	0.76	(0.70, 0.82)
**ACEF [5]**	0.68	(0.60, 0.75)	0.65	(0.58, 0.73)
**AGEF [6]**	0.63	(0.55, 0.70)	0.65	(0.58, 0.72)
**McCullough [8]**	0.49	(0.40, 0.57)	0.47	(0.37, 0.57)
**Mehran [7]**	0.68	(0.58, 0.76)	0.67	(0.59, 0.75)
**NCDR [9]**	0.65	(0.57, 0.73)	0.68	(0.61, 0.76)

*CI, confidence interval

**Table 4:** Sensitivity and specificity of indices for predicing AKI*.

Data Set	Sensitivity–Specificity	UT-AKI	Mehran	AGEF	ACEF	NCDR
**Derivation**	Sensitivity, Specificity (%)	80, 60	65, 63	67, 54	59, 76	28, 70
**Validation**	Sensitivity, Specificity %	84, 61	61, 62	71, 50	49, 68	55, 73

* The sensitivity and specificity of each predictive index is compared at specific thresholds or cut-points: UT-AKI (>0.1), Mehran (>5), AGEF (>1.48) and ACEF (>1.54). The McCullough score was not calculated as threshold values were not published.

We examined whether the predictive indices showed equal discrimination in classifying patients at risk for developing for mild (Stage 1) or more severe (Stage 2/3) AKI. For patients with Stage 1 AKI, the AUC for the UT-AKI (0.76) was higher than other indices (AUCs = 0.44–0.64) (S4 Table) For more severe AKI (Stage 2/3) all of the indices performed well (AUCs 0.70–0.80). For all of the indices, the mean scores rose significantly with the severity of the AKI (S5 Table).

As noted earlier, more severe AKI was associated with increased mortality and length of stay. To confirm the clinical validity of the UT-AKI score, we examined the relationship between UT-AKI scores and these these outcomes. Using pre-specified cut-points, higher UT-AKI scores were strongly associated with greater mean hospital stay and higher rate of in-hospital death (Table 5).

**Table 5:** Frequency of adverse clinical outcomes according to UT-AKI score.

	Score	AKI (N)	Hospital Death (N)	Mean Length of Stay in days (SD)
**Derivation Set**	0.02–0.09	5.1% (11)	2.2% (5)	3 (3)
	0.1–0.19	15.6% (19)***	3.3% (4)**	5 (5)***
	≥0.2	35.2% (31)***	10.2% (9)**	7 (7)***
**Validation Set**	0.02–0.09	3.6% (8)	1.3% (3)	4 (5)
	0.1–0.19	16.1% (19)***	0.8% (1)***	4 (4)***
	≥0.2	26.4% (24)***	13% (12)***	9 (11)***

**p<0.01

***p<0.001 compared to the low risk grroup scores ranging to 0.02–0.09

## Discussion

In this large, consecutive cohort of racially diverse patients, AKI as defined by modified RIFLE criteria, was associated with significant, 2.5-fold increases in the average hospital stay and nearly 6-fold increases in hospital mortality. AKI was a relatively common malady that affected 12.9% of patients. Although radiocontrast dye has been implicated in AKI in patients undergoing cardiac catheterization, we found no significant differences in the type of radiocontrast or the volume of contrast in those with or without AKI. There also was no significant difference in the frequency of AKI in patients receiving diagnostic angiography with or without PCI. Most patients with AKI had Stage 1, but 3% of patients had more severe, Stage 2/3 AKI. Patients with Stage 1 or greater AKI had significantly greater length of hospital stay and higher in-hospital mortality. Because of the clinical significance of Stage 1 or greater AKI, we derived and validated a predictive index to identify patients at risk. By ROC analysis, this UT-AKI index had a greater AUC than the other indices for predicting the development of AKI. The UT-AKI is available at https://www.uthsc.edu/cardiology/research/utaki.php.

A predictive index for AKI is necessary because a physician’s normal clinical assessment is not sufficiently accurate to predict risk. An advantage of the UT-AKI index vs. other indices was that it was derived on a randomly selected subset of a large consecutive series and then internally validated against a non-overlapping, random subset of data. Although not done in many studies, this type of internal validation enhances the generalizability of the index. The sensitivity and specificity of different classifications resulting from alternative ‘cut-off’ scores were also examined and found to provide consistent results. The UT-AKI performed well in predicting both mild (Stage 1) and more severe (Stage 2/3) renal injury. It had similar performance for identifying patients at risk after diagnostic angiography with or without PCI.

Calculation of an individual patient’s risk can be cumbersome and to simplify that process, previous indices have created point scores, at the expense of some loss in precision. In contrast, the UT-AKI fits a logistic regression model for each patient to calculate the probability that they will develop AKI given their specific clinical characteristics. This is made possible by a simple web-based interface without a loss in precision.

The predictive value of the UT-AKI index was compared to other indices using pre-defined classification scores or cut-off values that were taken, whenever possible, from the original studies, though not all studies were explicit in their cut-off values. The AUC of the UT-AKI index was greater than other indices with an AUC of 0.77 in the derivation set and 0.76 in the validation set. Considering the high mortality rate and the high rate of complications associated with AKI, it is crucial to classify correctly patients at risk [18]. As such higher sensitivity (for correctly identifying patients at risk for AKI), is relatively more important than specificity (correctly identifying who is not at risk). In general, the UT-AKI appeared to have a higher sensitivity in our patient population vs. other indices, whether analyses were performed using published cut-off values or the cut-off values derived for our patient population. (Table 4 and S3 Table).

In this study adverse hemodynamic factors were commonly associated with significant risk of AKI. Hypotension was 2–6 times more common in patients with Stage 1 or Stage 2/3 AKI. Patients with Stage 1 or Stage 2/3 AKI were three times more likely to have had intra-aortic balloon pump therapy than those who did not have AKI. The frequency of IABP therapy in this series reflects clinical practice prior to the publication of the IABP-SHOCK II trial, which demonstrated a lack for mortality benefit in patients with cardiogenic shock complicating acute myocardial infarction.[19] The incidence of AKI in our study (12.9%) is comparable to the incidence of 16.1% in the multicenter Horizons-AMI trial of STEMI patients and the incidence of 13.2% in the Mehran study of elective patients, although both of these studies examined contrast-induced nephropathy, defined as ≥0.5 mg/dL increase or a 25% relative rise an increase in serum creatinine. When we analyzed the outcomes of patients in this study, we found that patients meeting the definition of AKI had a significant increase in length of stay and mortality, whereas patients with contrast-induced nephropathy did not. In contrast to the Mehran study, in more recent studies we and others [20] have found no relationship between mean radiocontrast dye volume and AKI, which may reflect the ~40% lower mean contrast volume in this series and others [20] than was reported in the Mehran study [7]. Among these studies, the AGEF report was notable for its unusual, 2–3 fold lower incidence of AKI (5.2%). We also found that AKI was also associated with higher rates of adverse clinical events including major bleeding, death, re-infarction, target vessel revascularization or stroke [21]. The UT-AKI contains several hemodynamic variables (IABP, hypotension, LVEDP, LVEF) that independently contributed to improving the prediction of AKI. The strong associations between the magnitude of systolic dysfunction, the increase in left ventricular end diastolic pressures and the severity of AKI merit further study. The significant effect of these hemodynamic factors on the risk of AKI, and the lack of a significant association of contrast volume or type to AKI risk, suggests that the traditional label of contrast-induced nephropathy is inadequate to describe the factors that contribute to AKI in patients with STEMI. While the causes for AKI are not well understood, a biomarker substudy found that AKI was associated with activation of procoagulant molecules, decreases in endogenous anticoagulants, higher platelet activation, increased inflammation and diminished fibrinolysis [22].

### Limitations of the study

This was a retrospective analysis of a large, consecutive cohort of STEMI patients from a single institution with a large referral area. When compared to prospective studies, retrospective studies may underestimate the frequency of AKI. While the UT-AKI provides robust predictive information for AKI, it requires information that is generally available only after the PCI is performed; it was not possible to accurately predict AKI with the limited variables available to the physician prior to PCI. There are limitations in comparing prediction indices from previous studies, as there may be differences in cut-points and methodology, the definition and collection of variables, as well as the definition of acute kidney injury. In this study, splitting the data into derivation and validation data sets allowed development and evaluation of the UT-AKI; however, it may have diminished the potential power of the analyses, particularly for patients with severe, Stage 2/3 AKI. Although we performed an internal validation of the UT-AKI index, external validation will be necessary to determine how the index performs in different patient populations. Nearly all of the patients in this cohort had vascular access obtained via a femoral arterial approach; a recent study of acute coronary syndrome indicates that a transradial approach may reduce the risk of AKI [23]. The UT-AKI index comparably predicted risk in STEMI patients undergoing diagnostic angiography alone as well as in PCI patients, but its value in patients undergoing non-urgent, elective procedures has not been established. It is possible that clinical variables not included in our analyses may improve the classification of patients for AKI, but this will require further research in a different patient population.

### Summary

Currently, AKI affects one in about seven or eight patients referred for STEMI. A quick, web-based assessment such as the UT-AKI index may allow clinicians to more accurately predict which patients will develop AKI before it occurs, so that they may personalize subsequent therapies and diagnostic procedures to mitigate risk. For example, in a patient at increased risk, a physician may delay, modify or avoid subsequent therapies or testing that may increase the odds of renal failure (e.g., additional contrast studies, angiotensin inhibitors, routine coronary artery bypass surgery, nephrotoxic agents, etc.). Physicians may also monitor patients at high risk for AKI more closely, so that they might make early changes in medications or therapies to avoid complications associated with AKI, such as serious electrolyte disorders. Patients at risk also may be considered for other post PCI prophylactic therapies to prevent AKI. Finally, this index may prove useful in an enrichment strategy to identify patients with elevated risk for AKI for clinical trials of experimental therapies to improve the prevention of AKI and its associated complications.

## Supporting information

S1 Text Supplemental methods. (DOCX)

S1 Table Predictive indices for acute kidney injury. (DOCX)

S2 Table Prediction of CI-AKI by index classification score. (DOCX)

S3 Table Sensitivity and specificity of indices for predicting CI-AKI using Kolmogorov-Smirnov threshold scores. (DOCX)

S4 Table Predictive index AUC by stage of CI-AKI. (DOCX)

S5 Table Mean (SD) risk score by stage of CI-AKI. (DOCX)
