# Supplementary material for: A web-based tool to predict acute kidney injury in patients with ST-elevation myocardial infarction: Development, internal validation and comparison
Source: PLoS One. 2017 Jul 31;12(7):e0181658. doi: 10.1371/journal.pone.0181658 (PMC5536350; doi:10.1371/journal.pone.0181658)
Supplement: S1 Text — (DOCX) [file pone.0181658.s001.docx]

**S1 Supplemental Methods**

**Predictive Index Development.**

Sensitivity (also referred to as true positive rate or recall) is defined as the proportion of patients (correctly) predicted to develop AKI among those that actually do develop AKI. Specificity (or true negative rate) is defined as the proportion of patients predicted not to develop AKI among those who actually do not develop this condition. Because these measures are conditional on the respective groups that do and do not develop AKI, they are not prevalence dependent; this is in contrast to the accuracy of a classifier defined as the proportion of patients correctly predicted to develop or not develop AKI. These analyses focused on high specificity because in populations with comparatively few patients developing AKI, high specificity is relatively more important for achieving high accuracy, because the number of patients without AKI is much larger than those with AKI (i.e., specificity “acts” on the part of the patients that do not develop AKI). Two different approaches were taken to finding thresholds leading to binary classifiers with associated sensitivity and specificity. First, we adopted thresholds from previous studies. Second, we derived thresholds for all compared scores using the same optimality criterion based on the development data set; this allowed adaptation of the published models by deriving potential, patient-population-specific thresholds. This compensated for the potential implicit advantage of the UT-AKI score due to model development and parameter estimation in the same overall patient-population (but not the same subset of the data).
