# Supplementary material for: A web-based tool to predict acute kidney injury in patients with ST-elevation myocardial infarction: Development, internal validation and comparison
Source: PLoS One. 2017 Jul 31;12(7):e0181658. doi: 10.1371/journal.pone.0181658 (PMC5536350; doi:10.1371/journal.pone.0181658)
Supplement: S1 Table — (DOCX) [file pone.0181658.s002.docx]

**S1 Table: Predictive Indices for Acute Kidney Injury**

| **Predictive Index** | **Variables** |
| --- | --- |
| ACEF ^8^ | Age/EF + 1 (if pre-procedure serum creatinine >2) |
| AGEF ^9^ | Age/EF + 1 (if pre-procedure GFR <60) |
| McCullough ^11^ | exp{a}/(1+exp{a}) where a = 0.6011 – 0.1914(CrCl) + 1.6987(Diabetes*) + 0.0076(Contrast Volume) |
| Mehran ^10^ | 5 (if hypotension (SBP<80mmHg)) + 5 (if IABP) + 5 (if CHF) + 4 (if age greater than 75), + 3 (if anemia) + 3 (if diabetes) + 1 (for each 100 cc of contrast volume) + [4 (if serum creatinine >1.5mg/dl) or 2 (if eGFR 40-60) or 4 (eGFR 20-40) or 6 (eGFR <20) |
| NCDR ^4^ | 2 (if age 50-59) or 4 (if age 60-69) or 6(if age 70-79) or 8(if age 80-89) or 10(if age >90) + 18(if GFR <30) or 8(if GFR 30-45) or 3(if GFR 45-60) + 7(if Diabetes) + 4(if CHF) + 15(if STEMI) or 6(if nSTEMI/UA) + 16(if prior cardiac shock) + 10(if anemia) + 11(if IABP) + [variables not included in analysis due to incomplete data 11 (if prior 2 weeks HF) + 4(prior CVD) + 8(prior cardiac arrest)] |
| UT-AKI (this study) | Predictive probability directly obtained from the logistic regression model: exp{a}/(1+exp{a}) where a = – 3.748 + 1.288(IABP*) + 1.521(Hypotension*) - 0.032(LVEF) + 0.024(Age) + 1.293(CKD*) + 0.013(LVEDP) + 0.015(eGFR) |
| *categorical variable; yes = 1 | |
