# Supplementary material for: A web-based tool to predict acute kidney injury in patients with ST-elevation myocardial infarction: Development, internal validation and comparison
Source: PLoS One. 2017 Jul 31;12(7):e0181658. doi: 10.1371/journal.pone.0181658 (PMC5536350; doi:10.1371/journal.pone.0181658)
Supplement: S2 Table — (DOCX) [file pone.0181658.s003.docx]

**S2 Table: Prediction of CI-AKI by Index Classification Score***

|  |  | **UT-AKI >0.1** | | **Mehran >5** | | **AGEF >1.48** | | **ACEF >1.54** | | **NCDR>30** | |
| --- | --- | --- | --- | --- | --- | --- | --- | --- | --- | --- | --- |
| **Data Set** | **ACTUAL** | **No** | **Yes** | **No** | **Yes** | **No** | **Yes** | **No** | **Yes** | **No** | **Yes** |
| Derivation | No AKI | 60 (217) | 40 (147) | 64 (231) | 37 (133) | 54 (195) | 46 (169) | 76 (275) | 24 (89) | 70 (255) | 30 (109) |
|  | AKI | 20 (12) | 80 (49) | 34 (21) | 66 (40) | 33 (20) | 67 (41) | 41 (25) | 59 (36) | 72 (44) | 28 (17) |
| Validation | No AKI | 61 (234) | 39 (148) | 62 (238) | 38 (144) | 50 (192) | 50 (190) | 68 (260) | 32 (122) | 73 (277) | 27 (105) |
|  | AKI | 16  (8) | 84 (43) | 39 (20) | 61 (31) | 29 (15) | 71 (36) | 51 (26) | 49 (25) | 43 (23) | 57 (28) |

***Percentage is shown, N in parentheses**
