# Supplementary material for: A web-based tool to predict acute kidney injury in patients with ST-elevation myocardial infarction: Development, internal validation and comparison
Source: PLoS One. 2017 Jul 31;12(7):e0181658. doi: 10.1371/journal.pone.0181658 (PMC5536350; doi:10.1371/journal.pone.0181658)
Supplement: S3 Table — (DOCX) [file pone.0181658.s004.docx]

**S3 Table: Sensitivity and Specificity of Indices for Predicting CI-AKI Using Kolmogorov-Smirnov Threshold Scores**

| **Data Set** | **Sensitivity - Specificity** | **UT-AKI >0.12** | **Mehran >8** | **AGEF >1.4** | **ACEF >1.4** | **NCDR >22** |
| --- | --- | --- | --- | --- | --- | --- |
| Derivation | Sensitivity % | 72 | 54 | 71 | 69 | 67 |
|  | Specificity % | 67 | 79 | 53 | 70 | 37 |
| Validation | Sensitivity % | 77 | 45 | 73 | 55 | 78 |
|  | Specificity % | 67 | 75 | 49 | 63 | 38 |
