# Supplementary material for: A web-based tool to predict acute kidney injury in patients with ST-elevation myocardial infarction: Development, internal validation and comparison
Source: PLoS One. 2017 Jul 31;12(7):e0181658. doi: 10.1371/journal.pone.0181658 (PMC5536350; doi:10.1371/journal.pone.0181658)
Supplement: S4 Table — (DOCX) [file pone.0181658.s005.docx]

**S4 Table: Predictive Index AUC By Stage of CI-AKI**

| **Predictive Index** | **Stage 1** | **Stage 2/3** |
| --- | --- | --- |
| **UT-AKI** | 0.76 | 0.76 |
| **AGEF ^9^** | 0.60 | 0.75 |
| **ACEF ^8^** | 0.62 | 0.79 |
| **McCullough ^11^** | 0.44 | 0.70 |
| **Mehran ^10^** | 0.64 | 0.80 |
| **NCDR ^4^** | 0.64 | 0.76 |
