# Supplementary material for: A web-based tool to predict acute kidney injury in patients with ST-elevation myocardial infarction: Development, internal validation and comparison
Source: PLoS One. 2017 Jul 31;12(7):e0181658. doi: 10.1371/journal.pone.0181658 (PMC5536350; doi:10.1371/journal.pone.0181658)
Supplement: S5 Table — (DOCX) [file pone.0181658.s006.docx]

| **Predictive Index** | **CI-AKI** | | |
| --- | --- | --- | --- |
|  | **None** | **Stage 1** | **Stage 2/3** |
| **UT-AKI Risk Score** | 0.13 (0.12) | 0.25 (0.21)*** | 0.30 (0.23)*** |
| **AGEF ^9^** | 1.6 (1.0) | 1.9 (1.0)*** | 2.7 (1.8)*** |
| **ACEF ^8^** | 1.3 (0.8) | 1.5 (0.8)*** | 2.3 (1.6)*** |
| **McCullough ^11^** | 0.0048 (0.039) | 0.0056 (0.024)*** | 0.054 (0.14)*** |
| **Mehran ^10^** | 5.0 (4.1) | 7.6 (5.6)*** | 10.2 (4.9)*** |
| **NCDR ^4^** | 26.3 (10.6) | 32.1 (12.7)*** | 41.1 (14.3)*** |

**S5 Table: Mean (SD) Risk Score By Stage of CI-AKI**

***p<0.001 compared to no AKI as control.
